# The parafibromin tumor suppressor protein interacts with actin-binding proteins actinin-2 and actinin-3

**DOI:** 10.1186/1476-4598-7-65

**Published:** 2008-08-07

**Authors:** Sunita K Agarwal, William F Simonds, Stephen J Marx

**Affiliations:** 1 National Institute of Diabetes and Digestive and Kidney Diseases, NIH, Bethesda, Maryland, USA

## Abstract

**Background:**

Germline and somatic inactivating mutations in the *HRPT2 *gene occur in the inherited hyperparathyroidism-jaw tumor syndrome, in some cases of parathyroid cancer and in some cases of familial hyperparathyroidism. *HRPT2 *encodes parafibromin. To identify parafibromin interacting proteins we used the yeast two-hybrid system for screening a heart cDNA library with parafibromin as the bait.

**Results:**

Fourteen parafibromin interaction positive preys representing 10 independent clones encoding actinin-2 were isolated. Parafibromin interacted with muscle alpha-actinins (actinin-2 and actinin-3), but not with non-muscle alpha-actinins (actinin-1 and actinin-4). The parafibromin-actinin interaction was verified by yeast two-hybrid, GST pull-down, and co-immunoprecipitation. Yeast two-hybrid analysis revealed that the N-terminal region of parafibromin interacted with actinins. In actin sedimentation assays parafibromin did not dissociate skeletal muscle actinins from actin filaments, but interestingly, parafibromin could also bundle/cross-link actin filaments. Parafibromin was predominantly nuclear in undifferentiated proliferating myoblasts (C2C12 cells), but in differentiated C2C12 myotubes parafibromin co-localized with actinins in the cytoplasmic compartment.

**Conclusion:**

These data support a possible contribution of parafibromin outside the nucleus through its interaction with actinins and actin bundling/cross-linking. These data also suggest that actinins (and actin) participate in sequestering parafibromin in the cytoplasmic compartment.

## Background

Parafibromin is encoded by the *HRPT2 *gene located on chromosome 1q31[1] (OMIM# 145001 and 607393). *HRPT2 *inactivating mutations in the germline occur in the hyperparathyroidism-jaw tumor (HPT-JT) syndrome, characterized by hyperparathyroidism (parathyroid adenoma or carcinoma) (90%), fibrous-ossifying jaw tumors (30%), and bilateral renal cysts (10%) [1,2]. Germline *HRPT2 *mutations have also been detected in some cases of familial hyperparathyroidism [1,3,4], and biallelic somatic inactivation of *HRPT2 *is common in parathyroid tumor of HPT-JT and in sporadic parathyroid cancer [5-7].

Parafibromin has three possible nuclear localization signals; it is predominantly found in the nucleus [8-16] and in the nucleolus [17]. The C-terminal part of parafibromin (ORF = 531 amino acids) is homologous to yeast Cdc73p (ORF = 393 amino acids), an RNA polymerase II accessory protein [18]. Analogous to the presence of yeast Cdc73p in the yeast Paf1 protein complex, parafibromin was found in the human and *Drosophila *PAF1 protein complex [10,19-21]. Genetic interaction studies in *Drosophila *and protein interaction studies in mammalian cells have shown an interaction of parafibromin with β-catenin, mediating a functional association between Wnt signaling and the PAF1 complex [22].

The yeast two-hybrid system has been extensively used for the identification and analysis of protein-protein interactions [23,24]. To isolate parafibromin interacting proteins we conducted a yeast two-hybrid library screen, and we discovered an interaction between parafibromin and muscle actinins.

## Results

### Parafibromin interacting proteins

To identify proteins that interact with parafibromin, a yeast two-hybrid screen was performed with parafibromin as the bait. Because no parafibromin interacting proteins had been obtained by screening a human brain library (data not shown) and because of high-level expression of parafibromin in heart tissue [1], a human heart cDNA library was screened. From 10^6 ^yeast transformants, 76 colonies grew in selection medium; 36 of 76 were positive for the second reporter LacZ; upon retransformation 27 of 36 exhibited interaction with parafibromin. Sequencing of the inserts from the 27 plasmids revealed cDNAs encoding 6 different proteins (Table 1). There were 14 *ACTN2 *(actinin-2) sequences representing 10 independent clones, 5 *FHL2 *(four and a half LIM-domains 2) sequences representing 3 independent clones, 3 *DES *(desmin) sequences representing 3 independent clones, and one sequence each of *DMPK *(dystrophia myotonica protein kinase), *CLTCL1 *(clathrin heavy chain like-1) and *EEA1 *(early endosome antigen 1). All sequences were in-frame with the Gal4AD.

**Table 1:** Yeast Two-hybrid Library Screening

Gene Name	Entrez Gene ID	Description	Number of Colonies	Number of Independent Clones*
*ACTN2*	88	Skeletal muscle actinin, alpha-2 (actinin-2)	14	10
*FHL2*	2274	Four and a half LIM-domains 2	5	3
*DES*	1674	Desmin	3	3
*DMPK*	1760	Dystrophia myotonica protein kinase	3	1
*CLTCL1*	8218	Clathrin heavy chain like-1	1	1
*EEA1*	8411	Early endosome antigen 1	1	1

*ACTN2: 10 independent clones starting at amino acid (aa) 323, 327, 329, 333, 340, 360, 370, 377, 381 and 390 to the stop codon after aa 894; *FHL2*: 3 independent full-length clones differed in the length of the 5' UTR (63 bp, 75 bp, and 150 bp); *DES*: 3 independent clones starting after aa 142, 115, and 203 to the stop codon after aa 468.

Since actinin-2, FHL2, and desmin were represented by multiple independent library clones, they were analyzed further for interaction of their full-length coding regions with parafibromin. Full-length coding regions of all three were also positive for interaction with GalDBD-parafibromin (Table 2). Actinin-2 and FHL2 each belong to families of proteins; therefore, independent members of each family were tested for interaction with parafibromin (Table 2). Parafibromin selectively interacted with the skeletal muscle actinins (actinin-2 and actinin-3), but did not interact with the non-muscle actinins (actinin-1 and actinin-4). Among the four FHL family members, parafibromin only interacted with FHL2.

**Table 2:** Yeast Two-hybrid Interaction^† ^of Parafibromin Fused to Gal4DBD with Full-length Proteins Fused to Gal4AD

	Activation Domain Fusion	β-Galactosidase Assay
1	Actinin-1	-
2	Actinin-2*	++
3	Actinin-3*	+++
4	Actinin-4	-
5	α-actin	-
6	FHL-1	-
7	FHL-2	++
8	FHL-3	-
9	FHL-4	-
10	Desmin	++
11	β-catenin*	+

†Yeast strain MaV203 that contains the Gal4-LacZ reporter or EGY48/pSH-18 (for β-catenin) that contains the LexA-LacZ reporter was transformed with plasmids expressing Gal4DBD- or LexA-DBD-fusion proteins together with plasmids expressing Gal4AD-fusion proteins. Transformed yeast colonies selected in SD medium lacking Leu and Trp (Leu, Trp and Ura for EGY48/pSH-18) were scored for β-galactosidase activity in colony filter lift assays. Interaction is indicated by β-galactosidase activity level. -, +, ++, or +++ indicate no, low, medium or high levels of β-galactosidase activity, respectively. None of the Gal4AD fusions interacted with Gal4DBD alone or with Gal4DBD-p53 (aa 72–390 of mouse p53). The Gal4DBD-parafibromin did not interact with Gal4AD alone or Gal4AD-TAg (aa 87–708 of SV-40 T-antigen).

*Interaction was also positive with parafibromin fused to LexA-DBD (construct from [22]).

### GST pull-down and co-immunoprecipitation of parafibromin interacting proteins

**Figure 1:**
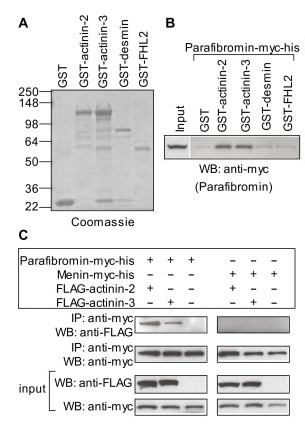
**Verification of yeast two-hybrid interaction of parafibromin with actinin**. (A) GST or GST-fusion proteins used for *in vitro *binding assay were analyzed by SDS-PAGE and stained with Coomassie Blue. On the left are shown molecular weights of the protein standards in kilodaltons. (B) GST pull-down assay: GST or GST-fusion proteins coupled to glutathione sepharose beads were incubated with whole cell protein extracts from HEK293 cells transfected with plasmid expressing parafibromin-myc-his. The beads were washed thoroughly and the bound parafibromin was detected by western blotting (WB) with an anti-myc antibody. Input corresponds to 1/40^th ^of the amount of protein extracts used for the pull-down assay. (C) Co-immunoprecipitation assay: Whole cell protein extracts from HEK293 cells transfected with plasmids expressing parafibromin-myc-his or menin-myc-his alone or together with FLAG-actinin-2 or FLAG-actinin-3 were immunoprecipitated with a rabbit anti-myc antibody. The immunoprecipitates were analyzed by western blotting (WB) with a mouse anti-myc (to detect parafibromin) or anti-FLAG (to detect actinin) antibody. Input panels show portions of protein extracts corresponding to 1/40^th ^of the amount used for each immunoprecipitation (IP), probed with anti-myc or anti-FLAG antibody.

To verify the *in vivo *finding in yeast of parafibromin interaction with actinins, desmin and FHL2, GST pull-down assays were performed (Figure 1). Protein extracts from HEK293 cells expressing parafibromin-myc-his were tested for binding to glutathione sepharose beads coupled with GST alone, or with GST-actinin-2, GST-actinin-3, GST-desmin, or GST-FHL2. Parafibromin was specifically associated with GST-actinin-2, GST-actinin-3, but not with GST alone, GST-desmin, or GST-FHL2 (Figure 1A and Figure 1B). Desmin and FHL2 were not analyzed further.

The interaction of parafibromin with actinins was further characterized *in vivo *by co-immunoprecipitation of actinin-2 or actinin-3 from protein extracts of HEK293 cells transfected with parafibromin-myc-his or menin-myc-his together with FLAG-tagged actinin-2 or actinin-3. FLAG-actinin-2 and FLAG-actinin-3 co-precipitated with parafibromin-myc-his but not with menin-myc-his (Figure 1C).

### Interacting regions of parafibromin and actinin

**Figure 2:**
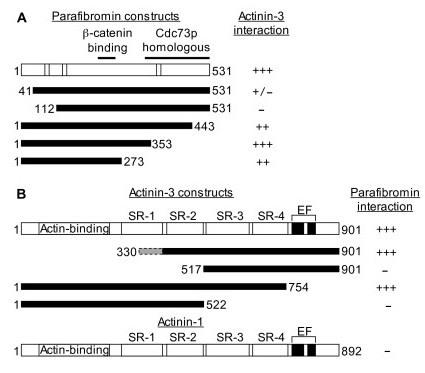
**Parafibromin and actinin interacting regions**. (A) Schematic of the 531 amino acid (aa) parafibromin protein. Striped regions are the 3 nuclear localization signals (aa 76–92, 125–139, and 393–409). The 2 black lines on top show the β-catenin interacting region (aa 218–263), and the Cdc73p homologous region (aa 343–531). Below are shown regions of parafibromin fused to the Gal4DBD that were tested for interaction with Gal4AD-actinin-3 by yeast two-hybrid assays as described in the footnote of Table 2. (B) Schematic of actinin-3 protein (901 amino acids). Following the N-terminal actin-binding region are 4 striped areas corresponding to the 4 spectrin repeats: SR-1 (aa 288–398), SR-2 (aa 408–512), SR-3 (aa 523–634), and SR-4 (aa 642–747). The two black areas near the C-terminus are the EF-hand domains (aa 764–793, and 800–828). Below are shown regions of actinin-3 fused to the Gal4AD that were tested for interaction with Gal4DBD-parafibromin by yeast two-hybrid assays as described in the footnote of Table 2. The dashed grey shaded area (aa 330–397) in the first actinin-3 construct corresponds to the location of the truncated N-terminus (aa 323, 327, 329, 333, 340, 360, 370, 377, 381 and 390) of the 10 independent actinin-2 clones, in-frame with the Gal4AD, isolated by yeast two-hybrid library screening. Also shown is the schematic of actinin-1 protein (892 amino acids) representing the two non-muscle actinins (actinin-1 and actinin-4) that do not interact with parafibromin. They show over 75% amino acid sequence identity with the muscle actinins (actinin-2 and actinin-3). Their domain composition and domain location is similar to the muscle actinins. However, the muscle actinins possess amino acid substitutions (not shown) in the EF hand domains that prevent calcium binding and render them insensitive to calcium. Calcium binding reduces the affinity of non-muscle actinins for F-actin [35]. Interaction is indicated by β-galactosidase activity level. -, +, ++, or +++ indicate no, low, medium or high levels of β-galactosidase activity, respectively. Activity level intermediate between 2 categories is indicated by a slash separating the 2 categories.

N- or C-terminal deletion constructs of parafibromin or actinin-3 were tested for interactions, in yeast two-hybrid assays (Figure 2). All three C-terminal truncations of parafibromin interacted with actinin-3, whereas the two N-terminal truncations of parafibromin did not interact with actinin-3. Each of the 10 yeast two-hybrid library clones (actinin-2) that was isolated as a parafibromin interactor was N-terminally truncated at amino acids 323, 327, 329, 333, 340, 360, 370, 377, 381 and 390 (Table 1). The N-terminus was located in the first spectrin repeat, and each interacted strongly with parafibromin. Further N-terminal truncations, deleting spectrin repeat 2 and 3 of actinin-3, resulted in impaired interaction with parafibromin. C-terminal truncation of 150 amino acids of actinin-3, deleting the 2 EF hand domains allowed interaction with parafibromin; but further C-terminal deletion of actinin-3, deleting spectrin repeats 3 and 4, prevented interaction with parafibromin.

### Actin filament bundling/cross-linking by actinin and parafibromin

**Figure 3:**
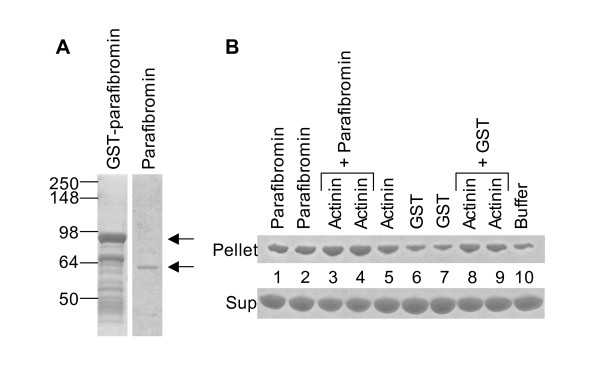
**Actin bundling/cross-linking by actinin and parafibromin**. (A) GST-parafibromin, and parafibromin separated from GST after thrombin cleavage was analyzed by SDS-PAGE and stained with Coomassie Blue. On the left are shown molecular weights of the protein standards in kilodaltons. Arrows show the bands corresponding to GST-parafibromin (upper arrow for the panel on the left) and parafibromin (lower arrow for the panel on the right). (B) G-actin was polymerized to F-actin. F-actin was incubated with rabbit skeletal muscle actinin, parafibromin (2 different preps) or GST (2 different preps) alone, or together as indicated, and centrifuged at 10,000 g for 15 minutes to sediment higher order structures of F-actin (bundles and/or cross-linked networks). The F-actin in the supernate (not bundled/cross-linked) and pellet (F-actin bundles/networks) fraction was analyzed by SDS-PAGE and stained with Coomassie Blue. Lane 10 (buffer) contains F-actin that was sedimented without any added GST, actinin or parafibromin, and therefore corresponds to the background level of F-actin bundles/networks in the pellet (similar background amount was also seen in the lane 6 and 7 marked GST). Increased actin, above background level, in the pellet fraction (lane 1, 2, 3, 4, 5, 8 and 9) indicates F-actin bundling and/or cross-linking.

Some actin-binding proteins organize actin filaments (F-actin) into higher order structures by F-actin bundling and/or F-actin cross-linking [25]. Actin bundling is the arrangement of parallel or anti-parallel linear arrays of actin filaments. Actin cross-linking causes the arrangement of actin into a network of orthogonal arrays. Actinins can bundle and cross-link actin filaments [26]. In a low speed (10,000 g) actin sedimentation assay, F-actin does not sediment in the pellet fraction unless it has been induced to form higher order structure by the action of bundling proteins or cross-linking proteins such as actinin [27]. Therefore, the effect of parafibromin on the actin bundling property of actinins *in vitro *was analyzed. In the actin sedimentation assay, F-actin bundles were found in the pellet fraction when F-actin was mixed with actinin alone or parafibromin alone, or actinin and parafibromin together; F-actin bundles were not found in the pellet fraction when F-actin was mixed with GST protein or buffer alone (Figure 3A and Figure 3B) or with RPA2 (data not shown). Thus, parafibromin did not inhibit the actin bundling property of actinin. However, parafibromin was itself capable of pelleting F-actin, indicating that parafibromin possessed some actin bundling (or actin cross-linking) activity.

### Differentiation induced cytoplasmic localization of parafibromin with actinins

**Figure 4:**
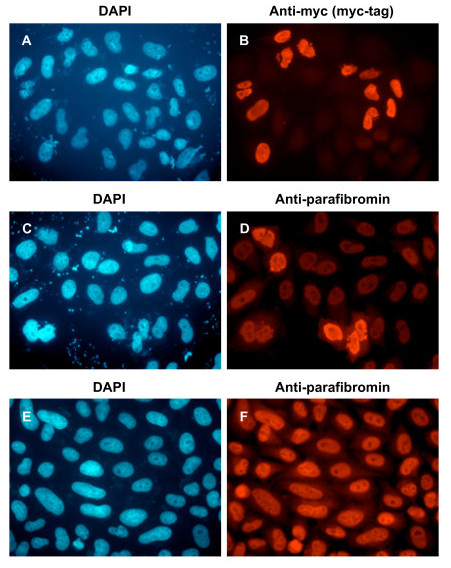
**Specificity of anti-parafibromin antibody (GRAPE) by immunofluorescence**. HeLa cells transfected with plasmid expressing parafibromin-myc-his (A, B, C, D) or untransfected (E, F) were analyzed by immunofluorescence with primary antibodies that recognize the myc-tag (B) or parafibromin (D, F), and secondary antibody conjugated to Texas Red, followed by DAPI staining (A, C, E). Magnification, 60×.

The specificity of the anti-parafibromin antibody GRAPE was analyzed in HeLa cells by detection of endogenous parafibromin or transfected parafibromin-myc-his. The immunofluorescence patterns detected by GRAPE or anti-myc antibody were identical, showing nuclear staining of cells transfected with parafibromin-myc-his (Figure 4). Also, nuclear staining representing endogenous parafibromin was specifically detected by GRAPE. HeLa cells transfected with parafibromin-myc-his showed morphologic changes associated with apoptosis (nuclear fragmentation and nuclear condensation) similar to those observed in cells transfected with a GFP-parafibromin construct [16].

**Figure 5:**
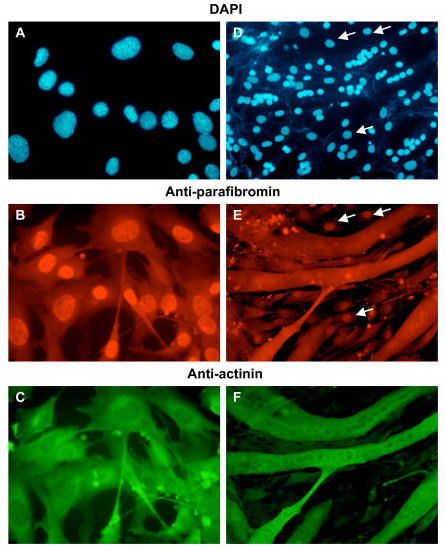
**Location of endogenous parafibromin and actinin in C2C12 myoblasts or C2C12 differentiated into myotubes**. C2C12 cells were analyzed by immunofluorescence with anti-parafibromin (GRAPE) antibody and anti-actinin antibody before (A, B, C) or after differentiation (D, E, F), and secondary antibody conjugated to Texas Red (to detect parafibromin) or fluorescein isothiocyanate (to detect actinin), followed by DAPI staining (A, D). Note that in panel E, along with myotubes there are some undifferentiated myoblasts that retain nuclear parafibromin staining (arrows). Magnification, 60× (A, B, C), or 10× (D, E, F).

Actinin-2 and actinin-3 are abundantly expressed in the cytoplasmic compartment when muscle precursor cells (myoblasts) are induced to differentiate into myotubes [28]. Therefore, localization of endogenous parafibromin and actinins was analyzed in the mouse myoblast cell line C2C12 that was undifferentiated or differentiated into myotubes. Parafibromin was predominantly nuclear in undifferentiated proliferating C2C12 cells; interestingly, in differentiated myotubes parafibromin was mainly located in the cytoplasmic compartment with actinins (Figure 5).

### Localization of endogenous parafibromin with transfected actinin in the cytoplasm

**Figure 6:**
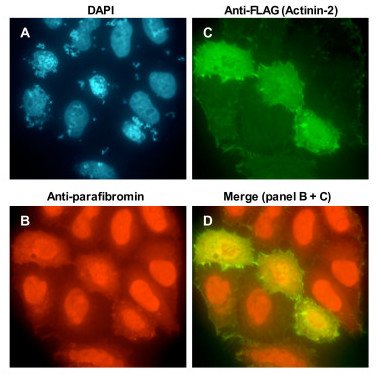
**Location of endogenous parafibromin in HEK293 cells transfected with actinin**. HEK293 cells transfected with plasmid expressing FLAG-actinin-2 were analyzed by immunofluorescence with primary antibodies that recognize parafibromin (GRAPE) or the FLAG-tag (actinin-2), and secondary antibody conjugated to Texas Red (B, to detect endogenous parafibromin) or fluorescein isothiocyanate (C, to detect transfected actinin-2), followed by DAPI staining (A). Note that in untransfected cells parafibromin is nuclear but in the 3 cells over-expressing FLAG-actinin-2, parafibromin is in the cytoplasm. Merged Texas Red (parafibromin) and fluorescein isothiocyanate (actinin-2) signal is shown in panel D. Magnification, 60×.

The co-localization of actinin and parafibromin in the cytoplasmic compartment in C2C12 cells stimulated to form myotubes could be unrelated to parafibromin-actinin interaction. Therefore, the ability of actinin to shift parafibromin from its predominantly nuclear location into the cytoplasm was analyzed by immunofluorescence in HEK293 cells. Transfection of a plasmid expressing actinin-2 in HEK293 cells caused accumulation of endogenous parafibromin in the cytoplasm (Figure 6). No change in morphology of HEK293 cells was evident upon co-localization of actinin and parafibromin in the cytoplasm.

## Discussion

One of the functional associations of parafibromin is with the PAF1 complex, involved in the regulation of transcriptional initiation and elongation in yeast, and based upon the presence of a C-terminal domain homologous to yeast Cdc73p [10,19-21]. This function of parafibromin is also connected to the Wnt signaling pathway by interaction with β-catenin [22]. In this interaction, parafibromin possibly recruits the PAF1 complex to β-catenin responsive genes thereby modulating the transcriptional output of β-catenin. In reporter assays, β-catenin mediated transcription could be regulated by *Drosophila *parafibromin (hyrax) but not by yeast Cdc73p [22]. In *Drosophila *with mutant hyrax alleles, human or *Drosophila *parafibromin could rescue the lethal phenotype [22]. The lethal phenotype of mutant hyrax alleles was not rescued by yeast Cdc73p. The presence of the N-terminal 350 amino acid region in human and *Drosophila *parafibromin that is not found in yeast Cdc73p suggests that, in addition to its Cdc73-associated function, parafibromin could possess other activities and/or functional associations.

Using the yeast two-hybrid approach, we report interaction of the N-terminus of parafibromin with muscle actinins. Multiple (n = 10) independent clones of actinin-2 were isolated in the yeast two-hybrid library screen, and the interaction was verified by other *in vitro *and *in vivo *methods suggesting a strong and significant interaction between parafibromin and actinin-2. Actinins (molecular mass ~100 kDa) are actin-binding proteins that are expressed in both muscle and non-muscle cells, and that cause bundling of actin filaments for cytoskeletal organization [29]. Actinins also serve to link the cytoskeleton, membrane proteins, and signaling proteins [30,31]. There are 4 alpha-actinins encoded by 4 different genes. The 4 proteins are highly homologous sharing 73–84% amino acid sequence identity [32]. Actinin-1 and actinin-4 are abundant in non-muscle cells, forming actin filament bundles such as stress fibers. They are associated with calcium dependent membrane attachment, and with cell-cell and cell-matrix adherent junctions [33]. In muscle cells, actinin-2 and actinin-3 are major structural components of striated muscle where they anchor actin filaments [34]. Actinin-2 is expressed in all muscle cells, whereas actinin-3 expression is limited to a subset of fast twitch skeletal fibers. In our yeast two-hybrid assays, parafibromin selectively interacted with the two muscle actinins (actinin-2 and actinin-3) but not at all with the non-muscle actinins (Table 2).

Actinins are composed of three distinct domains. An N-terminal actin-binding domain, a central rod-shaped domain with 4 internal spectrin-like repeats required for anti-parallel dimerization, and a C-terminal calmodulin-like domain containing a pair of calcium-binding EF hands [35] (Figure 2B). The EF-hands in non-muscle actinins, actinin-1 and actinin-4, are functional (calcium sensitive) whereas the EF-hands in muscle actinins, actinin-2 and -3 are non-functional (calcium insensitive). All the library clones of actinin-2 interacting with parafibromin were truncated at the N-terminus thus lacking the actin-binding domain and most of the first spectrin repeat of actinins. Further deletion of the N-terminus including the second spectrin repeat prevented interaction with parafibromin. Similarly, actinin-3 lacking its C-terminal EF-hand domains allowed interaction with parafibromin, but further C-terminal deletion including the third and fourth spectrin repeat (but retaining the first and second spectrin repeat) impaired interaction with parafibromin. Therefore, parafibromin likely interacts with the region of actinin containing spectrin repeats 2, 3 and 4 (Figure 2B). The region of highest variability among all actinins is in spectrin repeats 3 and 4 (not shown in Figure 2B) [36]. This could potentially explain why parafibromin interacts with muscle actinins and not with non-muscle actinins. Deletion of N-terminal 112 amino acids of parafibromin impaired actinin interaction. The N-terminal region of parafibromin minus its C-terminal Cdc73p homologous domain was sufficient for interaction with actinin (Figure 2A).

Actinins possess actin bundling and cross-linking activity [26,27,35]. In low speed actin sedimentation assays where actin filaments (F-actin) pellet only when they are arranged in higher order structures by cross-linking and/or bundling, just like actinin, to our surprise parafibromin was also capable of pelleting F-actin, indicating that parafibromin probably has inherent F-actin bundling or cross-linking activity. Moreover, despite parafibromin's predicted interaction with a part of the actinin dimerization region, parafibromin did not displace or disrupt actinin from F-actin bundles based on the observation that presence of parafibromin and actinin together appeared to slightly enhance the pelleting of F-actin (Figure 3B). This assay does not distinguish if the increased F-actin in the pellet fraction upon incubation of F-actin with actinin and parafibromin together was due to an increase in the number of F-actin bundles, or due to thicker bundles resulting from an increase in the number of F-actin filaments in individual bundles, or due to increased cross-linking. In yeast two-hybrid assays parafibromin did not interact with actin (Table 2). Parafibromin sequence analysis did not reveal any regions with significant homology to the several different actin-binding motifs that are found in actin-binding proteins [37].

Skeletal muscle myoblasts (such as modeled by C2C12 cells) can be induced to differentiate in mitogen-poor media to fuse and form multinucleate myotubes. This myogenic program is coupled to loss of cell proliferation and increase of differentiation. Actinins are important cytoskeletal structural proteins and their expression in the multinucleate myotubes increases upon induction of myotube formation [28]. Parafibromin's accumulation in the cytoplasm coincides with the inhibition of proliferation and with the activation of differentiation. The co-localization of parafibromin and actinin in the cytoplasm could be unrelated to their direct interaction. But the changed compartmentalization in HEK293 cells of endogenous parafibromin to the cytoplasm upon actinin transfection (Figure 6) indicates that the observation in C2C12 cells is likely related to parafibromin-actinin interaction. Parafibromin-actinin interaction might be directly important for mechanical strength and structure of the actin cytoskeleton, or the interaction could mediate other regulatory functions. Actin and actinin could play a role in regulating parafibromin levels in the nucleus for modulating parafibromin's transcription associated functions. The transcription associated tumor suppressor function of parafibromin could require its migration from the nucleus to cytoplasm as in C2C12 cells. Actin has been implicated in regulating gene transcription through several mechanisms, one of which is a direct role in controlling the nucleo-cytoplasmic distribution of transcription factors (Reviewed in [38]). Therefore, this interaction could be important for regulating the activity, and sub-cellular localization of parafibromin. Our observations in C2C12 cells also suggest other new aspects of parafibromin action such as a possible role in organization of the cytoskeleton and in myotube differentiation. We have investigated parafibromin-actinin interaction in muscle cells. The functional consequence of parafibromin-actinin interaction in cells affected in HPT-JT – parathyroid cells, bone cells, or renal cells – remains to be determined.

In C. elegans, RNAi against *HRPT2 *caused defects in the proper formation of polar bodies, pronuclei and mitotic nuclei indicating that *HRPT2 *is important for the proper completion of both meiosis and mitosis [39]. The phenotype analysis also suggested that the *HRPT2 *product is important for microtubule-based function in the early embryo. Perhaps, this function of parafibromin is also linked to its association with actinins.

## Conclusion

Our study does not directly address parafibromin's participation in the PAF-1 complex or the physiological function of parafibromin in the nucleus or cytoplasm, but the observation that interaction with actinins (and actin filament bundling/cross-linking) likely occurs when parafibromin accumulates in the cytoplasm suggests that a biological activity of parafibromin might be controlled by signals that promote its cytoplasmic accumulation. Further studies to recognize such signals and their consequences could provide insight into the functions of parafibromin in normal physiology and in tumorigenesis.

## Materials and methods

### Antibodies

Anti-parafibromin rabbit polyclonal antibody GRAPE was generated against a synthetic peptide (human parafibromin amino acid 262–299), and then affinity purified [16]. Mouse monoclonal anti-alpha-actinin clone BM-75.2 and mouse anti-FLAG M2 antibodies were from Sigma; rabbit anti-myc and mouse anti-myc antibodies were from Upstate. Rabbit IgG, mouse IgG, and HRP-conjugated secondary antibodies were from Santa Cruz Biotechnologies. Anti-rabbit secondary antibody conjugated to Texas Red and anti-mouse secondary antibody conjugated to fluorescein isothiocyanate were from Jackson ImmunoResearch Laboratories. Rabbit polyclonal affinity-purified peptide antibodies against actinin-2 and against actinin-3 were a gift from Dr. Neal Epstein of NHLBI, NIH.

### Mammalian cell culture and transfection

HEK293, HeLa, and C2C12 cell lines were obtained from American Type Culture Collection and grown in complete Dulbecco's modified Eagle's medium (DMEM containing high glucose, 2 μM glutamine, 1× concentration of antibiotic-antimycotic mix, and 10% fetal bovine serum) at 37°C in a humidified incubator containing 5% CO_2_. For differentiation of C2C12 myoblasts into myotubes, sub-confluent cells were grown for 4–6 days in differentiation medium (DMEM containing high glucose, 2 μM glutamine, 1× concentration of antibiotic-antimycotic mix, and 2% horse serum). Transient transfection was carried out with Polyfect (Qiagen) as per the manufacturer.

### Yeast two-hybrid assay

Yeast reporter strains for yeast-two-hybrid experiments were Y190 (Clontech), MaV203 (Invitrogen), or EGY48 (kind gift from Dr, Erica Golemis, Wayne State University). Matchmaker two-hybrid system II and the Mammalian Matchmaker two-hybrid assay kit were from Clontech.

The coding region of *HRPT2 *was amplified from human leukocyte marathon cDNA (Clontech) with primers containing restriction enzyme sites using Turbo Pfu (Stratagene), and cloned into the Gal4 DNA-binding domain (Gal4DBD)-fusion vector pAS2-1 in-frame with the Gal4DBD. All primers and PCR conditions in this study are available upon request.

pAS2-1-parafibromin was used as a bait to screen a yeast two-hybrid human heart cDNA library in the Gal4-Activation Domain (AD)-fusion vector pACT-2 (Clontech) by sequential transformation in Y190. The bait Gal4DBD-parafibromin did not have any transcription-activating property nor did it interact with Gal4AD alone or with Gal4AD-T-antigen. Transformed yeast were grown in 50 large (150 mm) petri dishes of SD -Trp, -Leu, -His selection media containing 20 mM 3-Aminotriazole (3AT) to control the leaky nature of the His+ phenotype. Colonies positive for growth were restreaked and tested for the second reporter LacZ (β-galactosidase (β-gal) activity) by colony filter lift assay (yeast protocols handbook, Clontech). Plasmids from colonies positive for growth in selection media and positive for β-gal activity (blue), corresponding to library plasmid expressing parafibromin interacting proteins, were retrieved by using the yeast DNA isolation system (Stratagene). These pACT-2 library plasmids were retransformed into yeast competent cells (MaV203) together with pAS2-1-parafibromin to confirm the protein-protein interaction. None of the library positives were self-activating nor did they interact with Gal4DBD-p53 or Gal4DBD alone. Plasmids that still were positive for parafibromin interaction after this testing were sequenced with vector primers at both ends of the insert.

Other constructs in the pAS2-1 or pACT-2 were generated by cloning in-frame inserts. Full-length coding region of actinin-1, actinin-2, actinin-3, actinin-4, desmin, FHL-1, FHL2, FHL-3, FHL-4, and α-actin was amplified using marathon cDNA (Clontech) from human skeletal muscle (actinin-2, actinin-3, desmin, FHL-1, FHL2, FHL-3, and α-actin), human brain (actinin-1 and actinin-4), or mouse testis (FHL-4), with primers containing restriction enzyme sites using Turbo Pfu (Stratagene), and cloned into pACT-2. Inserts containing various deletions from the N- and C- terminus of parafibromin or actinin-3 were generated by PCR amplification using appropriate primers, with pAS2-1-parafibromin or pACT-2-actinin-3 as the template, and cloned into pAS2-1 or pACT-2, respectively. For testing interaction of parafibromin deletion constructs, actnin-3 was used because the interaction of full-length actinin-3 with parafibromin was similar but slightly stronger than with full-length actinin-2. The LexA-DBD-parafibromin construct pCM031 and the Gal4AD-β-catenin construct pRH95 were provided by Dr. Christian Mosimann, University of Zurich [22].

Interaction of fusion-proteins expressed by the pAS2-1 and pACT-2 constructs were tested by transforming into yeast competent cells (MaV203). Yeast colonies growing on plates containing SD -Trp, -Leu selection media were tested for β-gal activity by colony filter lift assay. None of the pACT-2 constructs were self-activating nor did they interact with Gal4DBD-p53 or Gal4DBD alone. The interaction of parafibromin in LexA-DBD vector with β-catenin in Gal4-AD vector was tested in the yeast strain EGY48 using the LexA reporter pSH18-34 and SD -Trp, -Leu, -Ura selection media.

To verify the expression of fusion proteins in yeast, protein extracts were analyzed by western blot [40].

### GST pull-down assay

The full-length coding regions of actinin-2, actinin-3, desmin or FHL2 were sub-cloned from the yeast two-hybrid vector pACT-2 into the GST-fusion vector pGEX5X-2 (Amersham-Pharmacia). The coding region of parafibromin was amplified from leukocyte marathon cDNA and cloned into the myc-tag fusion vector pcDNA3.1(-)-myc-his-A (Invitrogen) in-frame with a downstream myc-his-tag. In order to generate an in-frame fusion of parafibromin at the C-terminus with the myc-his tag, the parafibromin stop codon was changed from TGA to AGA in the reverse primer.

GST or GST-fusion proteins (GST-actinin-2, GST-actinin-3, GST-desmin or GST-FHL2) were expressed in bacteria in the E. coli BL21-PRIL strain (Stratagene), and purified on glutathione sepharose beads (Amersham-Pharmacia) as described [40]. Similar amounts of GST alone, or GST-fusion proteins coupled to glutathione sepharose beads were incubated overnight at 4°C with whole cell protein extracts from HEK293 cells transfected with a plasmid expressing parafibromin-myc-his. The beads were washed thoroughly 5 times with the lysis buffer-1 (TBS containing 0.1% Triton X-100 and 1 mM DTT) that was used for preparing protein extracts, and resuspended in protein loading buffer. The bound parafibromin was detected by western blot with an anti-myc antibody.

### Co-immunoprecipitation

The full-length coding region of actinin-2 and actinin-3 was sub-cloned from the yeast two-hybrid vector pACT-2 into the FLAG-fusion vector pCMVtag2C (Stratagene). The plasmid construct expressing parafibromin-myc-his was described above, and the menin-myc-his construct was previously described [41]. Before their use in co-immunoprecipitation (Co-IP) experiments, the FLAG-actinin-2, FLAG-actinin-3, and parafibromin-myc-his expression was verified by transfecting HEK293 cells and analyzing whole cell protein extracts by western blot using specific antibodies (data not shown).

HEK293 cells were transfected with plasmids expressing FLAG-actinin-2 or FLAG-actinin-3 together with plasmids expressing parafibromin-myc-his or menin-myc-his. Whole cell protein extracts were prepared 48 h after transfection in lysis buffer-2 (20 mM Tris pH 8.0, 150 mM NaCl, 1 mM EDTA, 1% Triton X-100, 0.1% SDS, and 0.1% deoxycholate). Protein extracts were used for immunoprecipitation as described with 4 μg of rabbit anti-myc antibody [42]. Actinin and parafibromin were detected by western blot with anti- FLAG or mouse anti-myc antibodies, respectively.

### Actin sedimentation assay

The full-length coding region of parafibromin was sub-cloned from the yeast two-hybrid vector pAS2-1 into the GST-fusion vector pGEXIIT (Amersham-Pharmacia). The plasmid construct expressing GST-RPA2 was previously described [42]. GST, GST-parafibromin, or GST-RPA2 was expressed in bacteria in the E. coli BL21-PRIL strain (Stratagene) and purified on glutathione sepharose beads (Amersham-Pharmacia) as described [40]. GST-parafibromin or GST-RPA2 conjugated to glutathione sepharose beads was incubated in thrombin cleavage buffer (20 mM Tris pH 7.5, 150 mM NaCl, 2.5 mM CaCl2, and 1 mM DTT) with 10 U of thrombin (Sigma) per mg of GST-fusion protein to remove the GST tag. Thrombin was inactivated by adding a protease inhibitor cocktail (Roche Biochemicals) and 1 mM PMSF. The supernate containing parafibromin or RPA2, after thrombin cleavage, was passed through a benzamidine-sepharose column to remove the thrombin. The flowthrough was concentrated with an Amicon-50 (parafibromin) or an Amicon-10 (RPA2) concentrator. To obtain GST protein, glutathione sepharose beads loaded with GST were eluted with 50 mM Tris-HCl (pH 8) containing 10 mM reduced glutathione. To maintain similar treatment conditions GST was also incubated with thrombin, then with protease inhibitors, passed through a benzamidine-sepharose column, and concentrated with an Amicon-10 concentrator.

Low speed actin sedimentation assays used the Actin binding protein Biochem kit as per the manufacturer (Cytoskeleton). Briefly, rabbit skeletal muscle G-actin (monomeric globular actin) was polymerized to F-actin (polymerized filamentous actin). F-actin (36 μg) was mixed with actinin (10 μg) or parafibromin (4 μg) or GST (4 μg) or RPA2 (4 μg) or with thrombin cleavage buffer alone, or with actinin and parafibromin, or with actinin and GST, or with actinin and RPA2, and centrifuged at 10,000 g at room temperature for 15 minutes. The supernate was removed carefully and mixed with 12 μl of 5× protein loading buffer (supernatant fraction). The pellet was resuspended in 60 μl of 1× protein loading buffer (pellet fraction). 20 μl of the pellet fraction and 20 μl of the supernatant fraction were analyzed by SDS-PAGE, and the proteins were detected by Coomassie Blue.

### Immunofluorescence

Cells (HeLa, HEK293 or C2C12) were seeded on two-well chamber slides in complete DMEM and incubated overnight. To obtain differentiated C2C12 myotubes, sub-confluent cells were grown in differentiation medium for 4–6 days. Cells on chamber slides were fixed in PBS containing 4% paraformaldehyde for 20–30 minutes at room temperature and permeabilized with PBS containing 0.5% Triton X-100 for 5 minutes at room temperature. Conventional immunofluorescence was carried out as described [42]. Microscopy and photomicrography was performed with an epifluorescence microscope (Zeiss).

## Competing interests

The authors declare that they have no competing interests.

## Authors' contributions

SKA designed, performed, and analyzed all the experiments. SKA, WFS, and SJM participated in data interpretation and manuscript preparation. All authors read and approved the final manuscript.
